# Long COVID is associated with lower percentages of mature, cytotoxic NK cell phenotypes

**DOI:** 10.1172/JCI188182

**Published:** 2024-12-17

**Authors:** Tasha Tsao, Amanda M. Buck, Lilian Grimbert, Brian H. LaFranchi, Belen Altamirano Poblano, Emily A. Fehrman, Thomas Dalhuisen, Priscilla Y. Hsue, J. Daniel Kelly, Jeffrey N. Martin, Steven G. Deeks, Peter W. Hunt, Michael J. Peluso, Oscar A. Aguilar, Timothy J. Henrich

**Affiliations:** UCSF, San Francisco, California, USA.

**Keywords:** Immunology, Infectious disease, Innate immunity, NK cells

**To the Editor:** Following SARS-CoV-2 infection, approximately 5% of individuals develop long COVID (LC), defined as ongoing symptoms present at least 3 months after infection that are disruptive to everyday functioning. There is growing evidence that SARS-CoV-2 persistence is associated with LC and that people with LC have dysregulated adaptive immune function that may originate from or potentiate viral persistence (1). NK cells, a critical component of the viral innate immune response, can exhibit dysfunctional phenotypes during acute COVID-19, and these alterations may hinder clearance of SARS-CoV-2–infected cells. COVID-19 can also induce adaptive NK cell responses during acute infection (2, 3), but there is a critical lack of knowledge about NK cell phenotypes in the postacute phase and their relationship to LC.

We performed high-dimensional NK cell phenotyping on PBMCs collected 4 months following SARS-CoV-2 infection from people with LC (*N* = 21) and those who had prior COVID-19 but fully recovered (*N* = 14) in the UCSF Long-term Impact of Infection With Novel Coronavirus (LIINC) cohort (4) (Supplemental Methods and Supplemental Table 2). Samples were collected in the first year of the pandemic prior to the emergence of vaccination, antiviral treatment, or frequent reinfection, and most participants (74%) had mild/moderate outpatient COVID-19 (Supplemental Table 1; supplemental material available online with this article; https://doi.org/10.1172/JCI188182DS1). LC was defined as the presence of COVID-attributed symptoms at least 3 months following the initial infection, and severe LC was defined as the presence of more than 5 symptoms. All participants were confirmed to have consistent presence or absence of LC symptoms for up to 8 months. We examined associations between NK cell phenotypes (Supplemental Figure 2), demographic and LC clinical factors, and SARS-CoV-2 antigen persistence.

Age was similar across groups (Figure 1A), but a larger proportion of people with LC were female (Figure 1B), and those with LC were more symptomatic during acute infection (Figure 1C). There were no differences in SARS-CoV-2 IgG levels between LC and FR groups (Figure 1D). Whereas percentages of total CD56^+^ NK cells were similar across groups (Figure 1E), mature, cytotoxic CD56^dim^/CD16^+^ NK cell percentages were significantly lower in participants with LC compared with those who had fully recovered (*P* = 0.037, Figure 1F); a more pronounced significant reduction was observed in those with severe LC (*P* = 0.036; Figure 1G). CD56^dim^/CD16^+^ NK cells play an important role in clearing virus-infected cells from the body, and we observed a significant negative correlation between the percentage of these cells and the number of reported LC symptoms (Spearman’s *r* = –0.5, *P* = 0.022, Figure 1H). We also observed significantly lower CD56^dim^/CD16^+^ NK cell percentages in participants reporting any neurocognitive and gastrointestinal symptoms or fatigue compared with recovered controls, respectively (*P* = 0.037, *P* = 0.018, *P* = 0.041; Figure 1I). Participants with severe LC also had significantly lower percentages of CD56^dim^ NK cells expressing KIR2D (L1/S1/S3/S5) than the recovered group (*P* = 0.036; Figure 1G), but we did not identify a significant relationship between NK cell phenotypes and postacute plasma SARS-CoV-2 antigen detection (Supplemental Figure 1).

Given that the percentages of CD56^dim^/CD16*+* NK cells were significantly lower in female individuals than in male individuals (Figure 1J), we repeated comparisons of NK cell phenotypes, including only female individuals, and observed a significantly lower percentage of CD56*dim*/CD16*+* (47.1% vs 63.5%) and CD56^dim^/KIR2D(L1/S1/S3/S5)^+^ (16.2% vs 29.2%) NK cell populations in those with severe LC compared with those who fully recovered (*P* = 0.045 and 0.011, respectively; Figure 1K and Supplemental Figure 1). These data suggest that the differences observed implicate underlying factors between NK cell phenotypes and LC in addition to potential sex differences.

NK cells have been shown to acquire adaptive-like subsets and can be distinguished by their expression of NKG2C and loss of FcεR1γ and PLZF. Therefore, we performed a multidimensional analysis (Figure 1L) to define the relationship between adaptive NK cell phenotypes and LC as well as with other factors that may modulate LC, such as CMV seropositivity and the presence of EBV early antigen D IgG (a surrogate for recent EBV reactivation). Although we did not observe any significant differences in NK cell Uniform Manifold Approximation and Projection (UMAP) clusters with adaptive phenotypes between those with and without any LC phenotype, SARS-CoV-2 antigen detection, or EBV antibodies, we did observe a significant increase in the percentage of adaptive NK clusters in CMV IgG+ participants (*n* = 18) versus IgG– participants (*n* = 17) (*P* = 0.024; Figure 1M and Supplemental Figure 1).

In conclusion, we observed that the proportion of NK cells with a mature and cytotoxic phenotype was significantly lower in people with LC compared with those who fully recovered. These cells express CD16, an IgG receptor that mediates antibody-dependent cytotoxicity, and although we did not observe a direct association between postacute plasma SARS-CoV-2 antigen detection and these NK cell phenotypes, it is possible that reduced cytotoxicity during initial tissue infection or later clearance of persistently infected cells may be related to the development of LC. Alternatively, these data could suggest tissue redistribution of mature, cytotoxic NK cell subsets to tissues where antigen persists. Further study of the relationships among cytotoxic immune responses, viral persistence, and LC is urgently needed.

## Supplementary Material

Supplemental data

Supporting data values

## Figures and Tables

**Figure 1:**
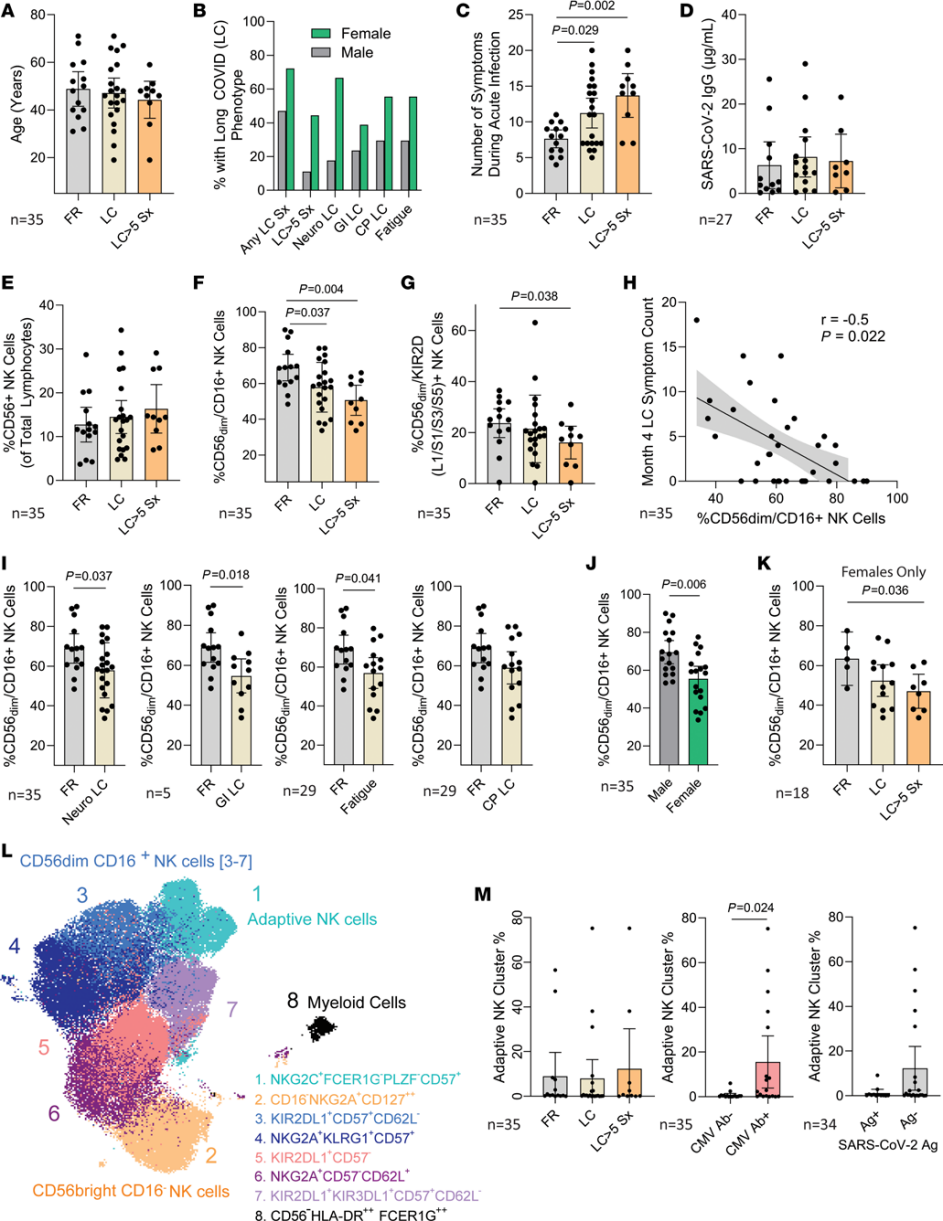
Relationships between NK cell phenotypes and long COVID. (**A**–**G**) Age (**A**), the percentage of male and female participants (**B**), the number of symptoms (sx) during acute infection (**C**), mean SARS-CoV-2 total anti-receptor binding domain (RBD) IgG levels (**D**), along with percentages of total CD56^+^ NK cells (**E**), CD56^dim^/CD16^+^ NK cells (**F**), and CD56^dim^ NK cells expressing KIR2D (L1/S1/S3/S5) (**G**) approximately 4 months following acute infection in those with long COVID (LC) and those who fully recovered. (**H** and **I**) Spearman’s correlation analysis of CD56^dim^/CD16^+^ NK cell percentages and the number of LC symptoms (**H**), and the percentage of CD56^dim^/CD16^+^ NK cells in participants with neurocognitive (neuro), gastrointestinal (GI), fatigue, and cardiopulmonary (CP) LC (**I**) are shown. (**J** and **K**) CD56^dim^/CD16^+^ NK cell percentages in male and female participants (**J**) and repeat CD56^dim^/CD16^+^ comparisons, including only female participants (**K**). (**L** and **M**) NK cell cluster UMAP (**L**) and the percentage of adaptive NK cell clusters by LC symptom group, CMV IgG serostatus, and presence or absence of SARS-CoV-2 spike or nucleocapsid antigen in blood after COVID-19 (**M**). Mean (bars) and 95% confidence intervals are shown. *P* values are from 2-tailed Mann-Whitney *U* testing (**I**, **J**, and **M**) or Kruskal-Wallis testing (**C**, **F**, **G**, and **K**).

## Version 1 12/17/2024

In-Press Preview

## Version 2 02/17/2025

Electronic publication
