# Supplementary material for: Long COVID is associated with lower percentages of mature, cytotoxic NK cell phenotypes
Source: J Clin Invest. 2024 Dec 17;135(4):e188182. doi: 10.1172/JCI188182 (PMC11827890; doi:10.1172/JCI188182)
Supplement: Supplemental data [file jci-135-188182-s050.pdf]

SUPPLEMENTARY MATERIAL

Table S1: Demographic and Clinical Data

	Long COVID	Recovered
Sample Size	N = 21	N = 14
Age in Years [median (range)]	46 (19-71)	49 (31-71)
Female birth sex [n (%)]	13 (61.9%)	5 (35.7%)
Race/Ethnicity		
White, Not Hispanic/Latino	10/20 (50%)	9 (64.3%)
Hispanic/Latino	8/20(40%)	2 (14.3%)
Black/African American	1/20 (5%)	1 (7.1%)
Asian	1/20 (5%)	2 (14.3%)
Hospitalized During Acute COVID-19	7 (33.3%)	2 (14.3%)
CMV IgG+[1]	10 (47.6%)	8 (57.1%)
EBV EAD IgG+	7 (33.3%)	6 (42.9%)
Detectable SARS-CoV-2 S or N Antigen[2]	8 (38.1%)	4/13 (30.8%)
Median Long COVID Symptom Count at Month 4 (IQR)	5 (4-9)	N/A
Median Days from Acute COVID-19 to Month 4 Sample Collection (IQR)	129 (122-135)	118 (112-124)

IQR=interquartile range, CMV=Cytomegalovirus, EBV EAD=Epstein Barr Virus Early Antigen D

[1] N=34

[2] Detected anytime following acute COVID-19 (S=spike, N=nucleocapsid)

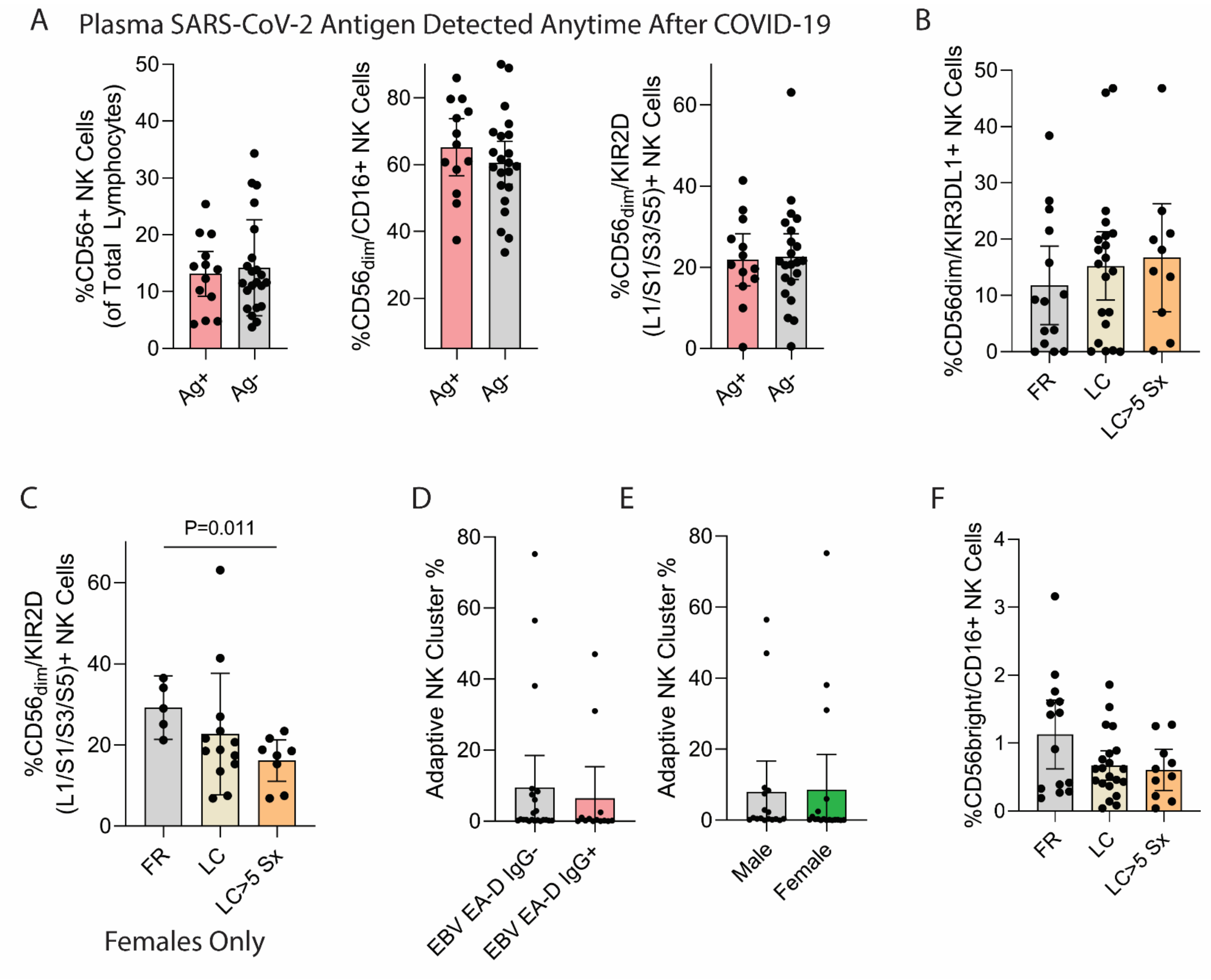

Fig S1. The percentage of total, mature/cytotoxic, and KIRD2(L1/S1/S3/S5)-expressing NK cells in participants with and without detectable SARS-CoV-2 Spike or Nucleocapsid antigen in plasma detected at any time following acute covid is shown in (A). The percentages of CD56dim NK cells expressing KIR3DL by LC group are shown in (B). The percentage of mature/cytotoxic NK cells by Long COVID (LC) symptom number in analyses including only female participants (C). Adaptive NK cell cluster percent in participants with detectable or undetectable EBV early antigen D (EA-D) IgG (D) and by sex/gender (E). LC symptom (sx) group includes participants with LC> 5 symptoms. The percentage of CD56birght/CD16+ NK cells by LC group is shown (F). Study N=35 for all analyses except for panel C (females only; n=18). Bars and lines represent mean values and 95% confidence intervals. P values from 2-tailed Mann-Whitney U tests or Kruskal-Wallis tests. FR = fully recovered at the M4 time point.

SUPPLEMENTARY METHODS

Sex as a Biological Variable: This study included both male and female participants (sex assigned at birth), and analyses were designed to identify sex-specific responses within Long COVID and fully recovered individuals.

Study Cohort and Sample Collections: Participants were enrolled in LIINC (www.liincstudy.org; NCT04362150) (1), a prospective observational study enrolling individuals with prior nucleic acid- or antigen-confirmed SARS-CoV-2 infection, regardless of the presence or absence of post-acute symptoms. All samples were collected in the first year of the pandemic, prior to the emergence of confounders such as vaccination, antiviral treatment, or frequent reinfection. Study details have previously been described (1). Briefly, at each visit, a trained interviewer administered an assessment of 32 physical symptoms newly developed or worsened since the COVID-19 diagnosis; symptoms that preceded the SARS-CoV-2 infection were not considered to represent Long COVID. The interviewer also collected detailed data regarding medical history, COVID-19 history, SARS-CoV-2 vaccination and SARS-CoV-2 reinfection were collected.

For this study, we selected participants who consistently met a case definition for LC based on the presence or absence of at least one symptom attributable to COVID-19 at least 4 months and 8 months following acute infection (NK cell and primary data collected approximately 4 months following acute infection/PCR positivity); this strategy was recently used in a publication examining immunologic signatures post-COVID (2).Our case definition is broadly consistent with both the World Health Organization and National Academies of Sciences, Engineering, and Medicine case definitions of LCAs in that study, participants were not deliberately matched by age and sex, but we ensured that there was overlap in the groups. Blood samples were collected between September 16, 2020 and April 6, 2021. All participants provided a post-COVID blood sample prior to ever having received a SARS-CoV-2 vaccination to avoid potential confounding effects of SARS-CoV-2 vaccination on our study; re-infections during this pandemic era were exceedingly rare and there were no known or suspected re-infections in the cohort. Specimens were collected approximately 4 months post-infection from all individuals. Peripheral blood mononuclear cells (PBMC) were collected in EDTA-coated tubes, isolated using

Fico-Hypaque, and cryopreserved in FBS with 10% FBS and stored in liquid nitrogen as previously reported (1). Double-spun plasma was aliquoted and stored at -80 C.

Study Approval: This study was approved by the University of California Institutional Review Board of the UCSF Human Research Protection Program (490 Illinois Street, San Francisco, CA 94143). All participants provided written informed consent.

Spectral Flow Cytometry: Following thawing, PBMCs were treated with FcR and monocyte blocker while stained with viability dye for 25 minutes. After washing, cells were stained with a cocktail of antibodies against surface markers for 30 mins at 4ºC (Supplementary Table 2). Cells were washed with FACS buffer before being permeabilized and fixed with BD Cytofix/Cytoperm (cat#554714). After washing, the intracellular markers were stained with our NK Cell panel of antibodies (Supp Table 1) for 30 minutes at 4℃ at a volume of 75uL. Stained cells were acquired on the 5 Laser Cytek Aurora Spectral Cytometer. Instrument laser settings were optimized using SpectroFlow beads, and then experimental settings were set up using unstained PBMCs along with single-stained reference controls. Unmixing errors were then corrected by spillover correction, and gating was done on FlowJo 10. After blind gating our cohort, the data was analyzed in depth on GraphPad Prism. An example of flow gating is shown in Fig S2.

NK cell Unsupervised Clustering Analysis: We utilized a conservative gating method to gate on NK cells ($CD45^{+}CD3^{-}CD14^{-}CD19^{-}CD56^{+}$ and/or $CD16^{+}$) from each patient. Equal number of NK cell events from each sample were concatenated and analyzed using the Uniform Manifold Approximation and Projection (UMAP) algorithm (FlowJo Plugin UMAP_R, version 4.1.1). Unsupervised clustering was performed on the expression of 10 highly differentially expressed markers using the FlowSOM algorithm which builds a self-organizing map and generates clusters based on consensus hierarchical clustering. Default parameters and a chosen number of 8 clusters were used. The levels of expression for the 10 highly differentially expressed markers across the clusters were visualized in a heatmap (FlowJo Plugin FlowSOM, version 4.1.0) (3).

SARS-CoV-2 Antigen Measurements: We used the Simoa® (Quanterix) single molecule array detection platform to measure SARS-CoV-2 antigens from spike, S1, and nucleocapsid (N) proteins as previously described in this participant cohort (4). The limit of detection (LOD) was determined as the background average enzyme per bead plus three times the standard deviation.

Statistics: Two-sided, non-parametric Mann-Whitney U tests were used to identify differences across various continuous variables for single comparisons and non-parametric Kruskal-Wallis with uncorrected Dunn's tests for multiple comparisons (given sample size limitations) using an alpha level of 0.05. All data were plotted for each graphical representation including mean (or median) values and 95% confidence intervals. Two-sided Spearman rank correlation analyses was used to determine r values and correlation significance between continuous variables. Linear regression lines with 95% confidence bands were used for graphical representation.

Data Availability: De-identified source data are available from the corresponding author upon request. Source data for all figures are included in the "Supporting Data Value" supplementary file.

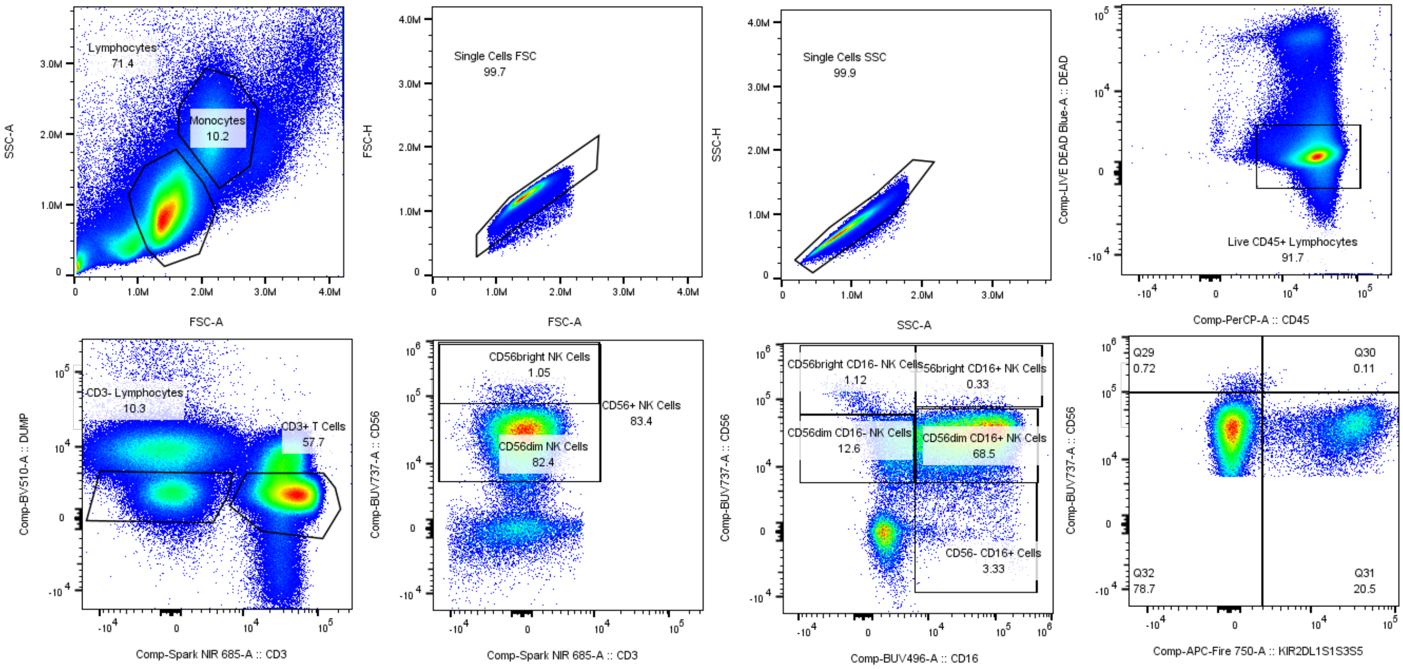

Fig S2. Example of flow cytometric gating of NK cell CD56, CD16 and KIR2DL(L1/S1/S3/S5) subsets.

Table S2. NK Cell Phenotyping Panel Antibodies

**Biomarker**	**Clone**	**Fluorophore**	**Catalog Number**
CD57	NK-1	BUV395	BD 567621
Live/Dead Blue		Viability UV	L23105 (TFS)
CD16	3G8	BUV496	BD 612944
KIR2DS2/L2/L3	DX27	BUV563	BD 748592
CD85J	GHI/75	BUV615	BD 752329
HLA-DR	G46-6	BUV661	BD 612980
CD56	NCAM16.2	BUV737	BD 612766
DNAM1	DX11	BUV805	BD 748427
IFN-g	4S.B3	BV421	BL 502532
NKp46	9E2	SB436	62-3359-42
CD69	FN50	Pacific Blue	BL 310920
CD161	HP-3G10	BV480	BD 748279
CD19	HIB19	BV510	BL 302242
CD14	63D3	BV510	BL 367124
CD4	OKT4	BV570	BL 317445
NKp30	P30-15	BV605	BL 325234
TNF-a	Mab11	BV650	BL 502938
KIR3DL1	DX9	BV711	BD 564102
NKG2D	1D11	BV750	BD 747025
CD38	HIT2	BV785	BL 303530
Fcer1g	polyclonal	FITC	FCABS400F (SA)
CD8	SK1	SparkBlue 550	BL 344862
CD45	2D1	PerCP	BL 368506
CD62L	SK11	BB700	BD 745995

Granzyme B	QA16A02	PerCP/Cy5.5	BL 372211
NKG2C	134591	PE	FAB138P-025 (RD)
PLZF	R17-809	PE/CF594	BD 565738
CD127	A01D5	PE/Cy5	BL 351324
IL-18Ra	H44	PE/Cy7	BL 313812
NKp80	5D12	APC	BL 346708
NKG2A	S19004C	AlexaFluor647	BL 375106
CD3	SK7	Spark NIR 685	BL 344862
CD107A	H4A3	APC/R700	BD 565184
KIR2DL1/S1/S3/S5	HP-MA4	APC/Fire 750	BL 339520
KLRG1	SA231A2	APC/Fire 810	BL 367732

BL = Biolegend; BD = BD Biosciences; TFS = Thermo Fisher Scientific; SA = Sigma-Aldrich; RD = R&D Systems

SUPPLEMENTARY REFERENCES

1. Peluso MJ, Kelly JD, Lu S, Goldberg SA, Davidson MC, Mathur S, et al. Persistence, Magnitude, and Patterns of Postacute Symptoms and Quality of Life Following Onset of SARS-CoV-2 Infection: Cohort Description and Approaches for Measurement. *Open Forum Infect Dis.* 2022;9(2):ofab640.
2. Yin K, Peluso MJ, Luo X, Thomas R, Shin MG, Neidleman J, et al. Long COVID manifests with T cell dysregulation, inflammation and an uncoordinated adaptive immune response to SARS-CoV-2. *Nat Immunol.* 2024;25(2):218-25.
3. Van Gassen S, Callebaut B, Van Helden MJ, Lambrecht BN, Demeester P, Dhaene T, et al. FlowSOM: Using self-organizing maps for visualization and interpretation of cytometry data. *Cytometry A.* 2015;87(7):636-45.
4. Peluso MJ, Swank ZN, Goldberg SA, Lu S, Dalhuisen T, Borberg E, et al. Plasma-based antigen persistence in the post-acute phase of COVID-19. *Lancet Infect Dis.* 2024;24(6):e345-e7.

ACKNOWLEDGEMENTS

LIINC Study Team Members:

We acknowledge current LIINC clinical study team members Brent Abel, Grace Anderson, Kofi Asare, Tyrine Bailey, Melissa Buitrago, Celina Chang Song, Alexus Clark, Avery Eun, Tony Figueroa, Diana Flores, Yuki Fudotan, Rebecca Hoh, Beatrice Huang, Megan Lew, Michael Luna, Kathleen Bellon Pizzaro, Douglas Robbins, Antonio Rodriguez, Dylan Ryder, Viva Tai, Julian Uy, Meghann Williams, and Andhy Zamora. We thank Sarah Goldberg, and Scott Lu for assistance with data management and Jessica Chen, Pahul Chhabra, Aidan Donovan, Marine Lyden, Sean Thomas, and Badri Viswanathan for assistance with data entry and review. We also acknowledge all former LIINC clinical, laboratory, and data team members. We thank the UCSF AIDS Specimen Bank for processing specimens and maintaining the LIINC biospecimen repository. We are grateful to Elnaz Eilkhani and Monika Deswal for regulatory support. We are also grateful for the contributions of additional LIINC leadership team members: Matthew Durstenfeld, Bryan Greenhouse, Isabelle Rodriguez-Barraquer, Rachel Rutishauser, and Ma Somsouk.

The authors are also grateful to the study participants and their medical providers.

Funding: TJH is supported on K24AI174971. MJP is supported on K23AI157875. TT is an NSF GRFP fellow supported on DGE-2445150 and T32GM136547. The LIINC clinical core is supported by the PolyBio Research Foundation, with additional funding from R01AI141003 (to TJH) and 1R01NS136197 (to MJP).

Conflicts of Interest

SGD reports consulting for Enanta Pharmaceuticals and Pfizer and reports research support from Aerium Therapeutics outside the submitted work. MJP has received consulting fees from Gilead Sciences, AstraZeneca, BioVie, Apellis Pharmaceuticals, and BioNTech and research support from Aerium Therapeutics and Shionogi, outside the submitted work. TJH reports consulting for Roche and received research funding from Merck outside the submitted work.

Author Contributions:

TT designed and conducted experiments, acquired and analyzed data, manuscript preparation (co-first author assigned given the work represents her graduate student project for which she helped design and implement the study)

AMB designed and conducted experiments, acquired and analyzed data, manuscript preparation (co-first author as research associated working with TT to conduct experiments and acquire data).

LG conducted experiments and acquired data

BL conducted experiments and acquired data

BAP conducted experiments and acquired data

EAF acquired data, managed participant enrollment, project management

TD acquired and managed data, data management

PYH designed research studies, manuscript writing

JDK designed research studies, manuscript writing

JNM designed research studies, cohort curation, manuscript writing

SGD designed research studies, cohort curation, manuscript writing

PWH designed research studies, provided data, manuscript writing

MJP designed research studies, clinical protocol oversight, cohort curation, acquired funding, manuscript writing

OAA conceived and designed research studies, conducted experiments, analyzed data, manuscript writing

TJH conceived the overall study, designed research studies, conducted experiments, analyzed data, acquired funding, manuscript writing
